# The Book World of Medicine and Science

**Published:** 1901-01-26

**Authors:** 


### THE BOOK WORLD OF MEDICINE AND SCIENCE.

ON SOME CIRRHOSES OF THE LIVER: BEING THE LUMLEIAN LECTURES FOR THE YEAR 1900, DELIVERED BEFORE THE ROYAL COLLEGE OF PHYSICIANS, LONDON. By WALTER BUTLER CHEADLE, M.A., M.D.Cantab. With Illustrations. (London: Smith, Elder, and Co. 1900. Price 5s.)

DR. CHEADLE has done well in publishing these lectures in the attractive and well-illustrated form in which they appear in this volume. Much as we know on the clinical side in regard to many of the symptoms associated with cirrhosis of the liver, cirrhosis itself is a thing in regard to which our knowledge is mostly derived from the post-mortem room. Hence our ideas regarding the prognosis of this disease have tended to be of a very gloomy character. Dr. Cheadle has shown in these lectures, however, that, notwithstanding the melancholy association of the word cirrhosis, the disease takes various forms, many of which may be treated hopefully and some successfully. The prognosis in cirrhosis must to a large extent hinge upon the stage in which it is first discovered, and the somewhat important observation is made by Dr. Cheadle that the symptoms by which its presence is indicated do not always arise quite in the order in which they might be expected. Ascites, for example—a symptom usually associated with an advanced stage of the malady—may now and again anticipate the ordinary term, and by giving early indication of the oncoming of cirrhosis may give such opportunity for remedial treatment that the patient may recover for a time—for months or even for years. The lectures deal chiefly with the three forms of the disease with which ascites in its most characteristic form is especially associated, namely, atrophic, hypertrophic, and syphilitic cirrhosis, and it is shown plainly enough that we have as yet by no means arrived at completeness of knowledge in regard to them. In the portion devoted to treatment many useful hints are given. The keynote of treatment is to be found in the attempt to preserve the *status quo* so far as the liver is concerned, and in relieving any difficulties which may arise in the bodily condition as the result of the hepatic inefficiency. Special attention is directed to the treatment of ascites, and in truth the main impression which one carries away after a perusal of these lectures is the lesson taught by Dr. Cheadle as to the advantage and necessity of early and repeated tapping carefully performed, whenever ascites causes any embarrassment of the viscera. Tapping in the very last stage of cirrhosis, he says, frequently hastens death, while a single paracentesis without repetition, or repeated only too late, when symptoms again become urgent, is of little service. In the successful cases which he records the patients were retained in hospital, or under constant medical care in private, until fluid ceased to return and health was fairly restored. Dr. Cheadle strongly protests against the maxim, "Tap and turn out," such a practice being, he says, futile and delusive. The book is certainly one to read, for it gives to the practitioner just that sense of hopefulness without which treatment can hardly expect to be successful.

ARSENICAL POISONING IN BEER DRINKERS. By T. N. KELYNACK, M.D., M.R.C.P., and WILLIAM KIRKBY, F.L.S. With sixteen illustrations. (London: Baillière, Tindall, and Cox. 1901. Price 3s. 6d. net.)

THIS is a topical book, exactly suited to the occasion, and it is admirably done. Dr. Kelynack and Mr. Kirkby are well known as having been among the first to investigate the serious outbreak of peripheral neuritis which has lately played such havoc among the beer drinkers in the Manchester district, and we have in this book, arranged in convenient form, a full account of the whole catastrophe. After a few pages of introduction of an historical and descriptive nature, we reach the main body of the book, which is divided into two portions, the first being clinical and the second chemical. The clinical portion consists of a capital review of the effects of chronic arsenical poisoning in the light of recent experience. It is truly clinical, for although books and opinions are referred to, all the descriptions are taken straight from the actual cases that have been observed in this epidemic, full details of many of these cases being given, and their appearance being well illustrated by a series of very nicely printed photographs of the conditions described. To those who wish to have in a handy form a fairly complete survey of the present position of our knowledge on the subject this book can be highly recommended, while those practising in and around the districts in which this form of poisoning is current will find it an almost necessary companion in their daily work. The second part of the book, which occupies a much smaller space than the clinical portion, is chemical, and is devoted to a description of the chemical problems involved and the special methods required in the detection of arsenic in beer and glucose. Of the various methods employed from time to time for the detection of arsenic Mr. Kirkby prefers that of Gutzeit, which indicates with great certainty the presence of even very minute quantities. The chief difficulty, indeed, with this test arises from its extreme delicacy, but if checked by using lead as well as mercury papers, so as to make sure of the absence of any excess of sulphur, it is shown to be a reliable test for arsenic. Medical practitioners, however, will find the chief interest of this book in the clinical portion, and in the striking photographic representations of the paralyses, the wastings, and the pigmentations which have been caused by the consumption of arseniated beer. In regard to the pathology of the affair it is well to note what Dr. Kelynack says of the extent to which arsenic affects the tissues. It is not a mere matter of "neuritis," for, as he points out, changes have been observed also in the ganglia cells of the anterior horns of the spinal cord; and in regard to the other tissues of the body "a widespread degeneration is characteristic. Most of the organs and muscles of the body present more or less fatty degeneration." The extensive prominence which has been given to the term "neuritis" in connection with this epidemic makes it desirable to emphasise the fact that the injurious effects of the poison have by no means been confined to the nervous system. Even the skin lesions, which some might feel inclined to consider "trophic" and secondary to nervous disturbance, are shown to have been in some cases associated with a very definite and measurable deposit of arsenic in the desquamating epidermis.

